# A promising future for endometriosis diagnosis and therapy: extracellular vesicles - a systematic review

**DOI:** 10.1186/s12958-022-01040-y

**Published:** 2022-12-21

**Authors:** Simon Scheck, Emily S. J. Paterson, Claire E. Henry

**Affiliations:** 1 Department of Obstetrics, Gynaecology and Women’s Health, University of Otago, Wellington, New Zealand; 2 Department of Obstetrics and Gynaecology, Wellington Hospital, Capital and Coast District Health Board, Wellington, New Zealand

**Keywords:** Endometriosis, Extracellular vesicles, Exosomes

## Abstract

Endometriosis is a chronic, inflammatory gynaecological disease that can have severe negative impacts on quality of life and fertility, placing burden on patients and the healthcare system. Due to the heterogeneous nature of endometriosis, and the lack of correlation between symptom and surgical disease severity, diagnosis and treatment remain a significant clinical challenge. Extracellular vesicles (EVs) are biologically active particles containing molecular cargo involved in intercellular communication, that can be exploited for diagnostic and therapeutic purposes.

We systematically reviewed studies exploring EVs and their role in endometriosis, specifically addressing diagnostic and therapeutic potential and current understanding of pathophysiology. Five databases (Pubmed, Embase, Medline, Web of Science, Google Scholar) were searched for keywords ‘endometriosis’ and either ‘extracellular vesicles’ or ‘exosomes’.

There were 28 studies included in the review. Endometrium derived EVs contribute to the development of endometriosis. EVs derived from endometriosis lesions contribute to angiogenesis, immunomodulation and fibrosis. Such EVs can be detected in blood, with early data demonstrating utility in diagnosis and recurrence detection. EV isolation techniques varied between studies and only eight of twenty-eight studies fully characterised EVs according to current recommended standards. Reporting/type of endometriosis was limited across studies. Varied patient population, type of sample and isolation techniques created bias and difficulty in comparing studies.

EVs hold promise for improving care for symptomatic patients who have never had surgery, as well as those with recurrent symptoms after previous surgery. We encourage further EV research in endometriosis with the inclusion of rigorous reporting of both the patient population and technical methodology used, with the ultimate goal of achieving clinical utility for diagnosis, prognosis and eventually treatment.

**Supplementary Information:**

The online version contains supplementary material available at 10.1186/s12958-022-01040-y.

## Background

Endometriosis is a heterogeneous condition with wide variation in reported prevalence [1], estimated to affect 10% of women worldwide (190 million women globally) [2]. The condition is defined by the presence of endometrial-like glands and stroma in locations outside of the endometrial cavity; and can manifest with dysmenorrhoea, subfertility and a heterogeneous range of other symptoms with little correlation between disease and symptom severity [3].

The diagnosis of endometriosis at present is reliant on clinical history and examination, ultrasound, and ultimately laparoscopic surgery and biopsy [3, 4]. Whilst endometriomas and deep infiltrating endometriosis can be diagnosed with increasing reliability by ultrasound [3], the more common manifestation of superficial peritoneal disease is poorly predicted without surgery [5].

Around half of women who have clinically suspected endometriosis undergo surgery to confirm the diagnosis [6]. Clinical suspicion of endometriosis is an inconsistent predictor of disease; of women undergoing laparoscopy for suspected endometriosis, 18–77% have surgically confirmed disease [7], although recent evidence suggests that the rates of microscopic endometriosis in women with “negative laparoscopy” may be up to 39% [8]. There is a strong need for a reliable non-invasive diagnostic tool to reduce the need for laparoscopy to diagnose or exclude endometriosis.

Management of endometriosis is generally divided into conservative, hormonal and surgical treatments. There is need for non-hormonal, non-surgical treatment options for endometriosis, especially in those actively trying to conceive. Surgical management is frequently utilised; however, due to vast heterogeneity of the literature, the benefits of surgery are debated and appear to be dependent on surgeon, operating technique, symptoms, disease stage and multiple other factors [3, 9]. Recurrence is common after surgery, with increasing risk of recurrence over time. Treatment after surgery to prevent recurrence at present is largely reliant on suppression of menstruation, with limited efficacy [3].

Extracellular vesicles (EVs) are non-replicating particles delimited by a lipid bilayer that are released by all cells. EVs carry a variety of cargo involved in extracellular communication, altering the recipient cell phenotype [10]. EV is an umbrella term for different secreted particles secreted that differ by size and biogenesis, generally between 40 and 4000 nm in size, including apoptotic bodies, microvesicles and exosomes [11]. EVs contain bioactive cargo such as selective packaging of proteins, mRNA and miRNA species, which are reflective of the cell of origin and can provide insight into the physiological state of the tissue. EVs are abundant in bodily fluids making them prime candidates for use as “liquid biopsy”, having been successfully isolated from blood, urine and saliva [12]. There is hope that EVs may replace tissue diagnosis as a reliable and non-invasive diagnostic tool for many pathological processes [13].

This diagnostic and prognostic potential has led to an escalation in EV research in the past years [13]. As such, the International Society for Extracellular Vesicles (ISEV) was formed in 2012, providing reporting guidelines to ensure scientific rigour, reproducibility and validity of EV research. The minimal information for studies of extracellular vesicles (MISEV2018) guidelines proposed by the ISEV recommend all EV studies report detailed sample collection, EV isolation methodologies and EV characterization [14]. Knowledge of the role of EVs in endometriosis has rapidly expanded in recent years. These recent advances explore both diagnostic and therapeutic potential, as well as giving an ever-increasing understanding of the underlying pathophysiology. With longstanding multiple theories of endometriosis pathophysiology including Sampson’s theory of retrograde menstruation [15], metaplastic theory and others [16], EVs have provided a plausible connecting link between these hypotheses [16, 17]. It has been established that EVs released from the endometrium of people with endometriosis differ from those without endometriosis [18–20]; and retrograde flow of such EVs has been confirmed by their identification in peritoneal fluid [17]. It has been hypothesised that these EVs potentiate migration and implantation of endometrial cells shed during retrograde menstruation, as well as manipulating the immune response to avoid their destruction, bearing many similarities to their role in cancer [17]. Furthermore, EVs released from newly implanted ectopic endometrial cells (i.e. endometriosis lesions) have been identified, and hypothesised to drive angiogenesis and neurogenesis, creating vascular, proliferative and potentially painful lesions [17]. These processes are most plausible and relevant for peritoneal deposits, but may also play a role in endometriomas, deep infiltrating endometriosis lesions and even rarely reported distant lesions (e.g. lung, brain) [16]. As peritoneal endometriosis is the most challenging to diagnose without surgery, EVs hold potential as a non-surgical diagnostic tool for these patients. For EVs to be of diagnostic value, they need to be both unique to the process of endometriosis and easily detectable in non-invasive samples (such as blood, urine, cervical secretions or potentially even stool samples [21]). If both criteria could be met, especially for patients with disease not detectable on ultrasound, the need for laparoscopy to diagnose or exclude endometriosis could be avoided, and it may be possible to gain information about disease severity pre-operatively for those needing surgery. In addition, such EVs could provide therapeutic targets for non-hormonal and non-surgical treatment; potentially modifying disease progression and recurrence.

Here we present a systematic review of studies exploring EVs and their role in endometriosis, specifically addressing diagnostic and therapeutic potential and the contribution to the current understanding of pathophysiology. We explore which bodily fluids these EVs have been reported in, how the EVs were detected and their proposed function.

## Methods

A systematic review was performed following the 2020 PRISMA guideline [22]. Five databases (Pubmed, Embase, Medline, Web of Science and Google Scholar [limited to 50 results by relevance]) were searched for keywords “endometriosis” and either “extracellular vesicles” or “exosomes” on 24/3/22 (see Additional File 1 for search strategy). No further studies were identified from citations during the literature review. Details of the protocol for this systematic review were registered on PROSPERO and can be accessed at https://www.crd.york.ac.uk/PROSPERO/display_record.php?ID=CRD42022302637.

### Inclusion and exclusion criteria

Articles were included if they were in English, peer reviewed, and assessed samples of extracellular vesicles in subjects with endometriosis (with or without controls). Human and animal studies were included. Reviews and conference abstracts were excluded.

### Data extraction

Studies were assessed for the following information: human or animal studies; source of bodily fluid to obtain EVs; isolation technique to identify EVs; target within EVs being studied; implications for diagnosis, treatment and pathogenesis of endometriosis; and limitations. Data from reports were extracted according to these headings (as shown in Table 1), with all three authors contributing independently to the data extraction and collation. No automation tools were required, and no further data was requested from included study authors.

**Table 1:** Key characteristics of studies of extracellular vesicles in endometriosis

Study	Species (n of endometriosis patients)	Controls (n)	EV Sample Source	EV Isolation technique	Characterisation requirements met^a^	EV cargo	Implications			Limitations
							**Diagnostic**	**Therapeutic**	**Pathogenesis**	
Chen 2019 [23]	Human (*n* = 6)	Human (*n* = 3)	Peritoneal fluid	UC	No	Small RNA species (seq). RT-qPCR: miR-130-3p validated, miR-1908-5p not statistically significant	Not discussed, but miR-130-3p a potential diagnostic target	Not discussed	EVs contain miRNAs that may contribute to immunomodulation and cell proliferation in endometriosis	Limited numbers. Only validated 2 miRNAs via RT-qPCR. No description of UC methods
Feng 2020 [24]	Human (*n* = 6)	None	CCM of primary umbilical cord derived MSCs	Exosome kit (#E1310; Bioruo)	Partially	No investigation	Not discussed	Not discussed	EVs from MSCs may enhance cell migration of endometrial cells	Limited numbers. EV source and endometriosis tissue from different patients
Harp 2016 [19]	Human (*n* = 5)	Human (*n* = 5)	CCM of ESCs derived from endometrial biopsies and endometriosis lesion biopsies	UC	Yes	miRNAs (seq). RT-qPCR validation of miR-21-5p (*p* < 0.0001), miR-126-5p not significantly different	Not discussed, but miR-21-5p a potential diagnostic target	Not discussed	EVs from endometrium in endometriosis patients may promote angiogenesis	Limited numbers
Hsu 2021 [25]	Human (*n* = 3)	Human (*n* = 3)	CCM of ESCs derived from endometrioma and endometrial biopsy	UC	Yes	Annexin A2 (ANXA2)	Not discussed, but potential diagnostic target	Not discussed	EVs from endometriosis lesions not found in endometrium may promote cell migration and angiogenesis	Limited numbers. May be specific to endometrioma only. Controls are endometrial biopsies from patients with endometriosis
Huang 2022 [26]	Not stated	Not stated	Endometrial tissue and normal human serum	UC	Partially	No investigation	Not discussed	Downregulation of miR-301a-3p reduces macrophage activity miR-301a-3p inhibition reduced PI3K expression and promotes PTEN expression in macrophages	Overexpression of miR-301a-3p in endometriosis lesions increases macrophage activity EVs from endometrial tissue increase M2 macrophage polarisation, through upregulation of PI3K and downregulation of PTEN	Study does not specify number of participants. Limited description of tissue source and methods. Compared tissue from lesions with serum from controls. Imply EV miR-301a-3p is the mechanism for EV induced changes in macrophage activity without demonstrating EVs contain miR-301-3a
Khalaj 2019 [18]	Human (*n* = 6)	Number not specified	Endometriosis lesions, endometrial biopsy, peritoneal fluid and plasma	MiRCURY exosome isolation kit (#300,102; Exiqon Inc)	Partially	Small RNA species (seq). RT-qPCR validation of miR-27a, miR-200c, miR-200a-5p, miR-375 and let-7a. miR–30d-5p, miR-100, miR-136 and miR-193b, miR-206 not significantly different.	Potential diagnostic targets: Unique EV miRNA signatures differing between: endometrium from endometriosis patients and controls, plasma from endometriosis patients and controls, and matched endometrium and endometriosis lesions. miR-30d-5p, miR-27a-3p and miR-375 network unique to endometriosis	Not discussed	Increased angiogenesis, cell growth and pro-inflammatory effects in presence of EVs from endometriosis lesions	Limited numbers. Control samples not clearly stated in methods
Lee 2021 [27]	Human (*n* = 45)	Human (*n* = 45)	Peritoneal fluid	UC	Partially	Microbiota composition of EVs	Not discussed, but potential diagnostic targets	Not discussed	Microbiome of peritoneal fluid and/or genital tract may influence development of endometriosis	May be specific to women with endometrioma
Li 2020 [28]	Human (serum *n* = 48)	Human (serum *n* = 21)	CCM of ESCs from endometrium and endometriosis lesions, serum	CCM: ExoQuick-TC Exosome Precipitation Solution (#EXOTC10A-1, SBI) Serum: SEC	Yes	VEGF-C	Serum EV VEGF-C provided sensitivity of 81.3%, and specificity of 71.4%	Potential therapeutic target (e.g. with levatinib)	EV secreted VEGF-C induces lymphangiogenesis and pro inflammatory microenvironment to promote endometriosis progression	Number of participant samples used for CCM EV assays unclear
Li 2021 [29]	Human (*n* = 23) Mouse (*n* = 18)	Human (*n* = 10) Mouse (*n* = 9)	CCM of M1 macrophages from endometrial biopsy	UC	Partially	Not investigated		Treatment of endometriosis with M1NVs (M1 macrophage derived EVs) safe in mouse model	M1NVs inhibit angiogenesis, migration and invasion of endometriosis, via reprogramming of M2 macrophage phenotype	EV cargo not investigated – mechanism of reprogramming unknown
Liu 2021 [30]	Human (*n* = 50)	Human (*n* = 50)	CCM of primary peritoneal macrophages isolated from peritoneal fluid	UC	Yes	lncRNA CHL1-AS1	Not discussed, but a potential diagnostic target	lncRNA CHl1-AS1 a potential therapeutic target	Peritoneal macrophage derived EVs downregulate endometrial cell apoptosis, and enhance proliferation and migration via lncRNA CHl1-AS1-miR-610 and MDM2 pathway	
Munrós 2017 [31]	Human *N* = 65	Human *N* = 33	Plasma	No isolation method from plasma	No	Total circulating microparticle (cMP). Circulating Microparticle Tissue Factor (cMP-TF)	cMP only elevated in patients with DIE or endometrioma	Not discussed, but potential target	Not discussed	Incorrect nomenclature. No description of EV isolation methods or EV characterisation. High likelihood of contamination from plasma lipids may affect cMP quantification and cMP specific TF analysis with ELISAs.
Muth 2015 [32]	Rhesus Macaque (*n* = 1)	Rhesus and pig tailed macaques (*n* = 2)	Cervicovaginal swab and lavage	UC	No	Not investigated	Cervicovaginal swab a potential source of EVs for diagnosis	Not discussed	EVs in endometriosis samples were less than healthy controls	Limited numbers. Limited reporting of EV isolation methodology.
Nazri 2020 [33]	Human (*n* = 22)	Human (*n* = 6)	Peritoneal fluid	SEC	Yes	Proteomics; PRDX1, ANXA2, ITIH4, H2A type 2-C and tubulin α-chain unique to endometriosis	Potential diagnostic target	Potential therapeutic target	Unique proteins in endometriosis samples	Limited numbers
Qiu 2019 [34]	Human (*n* = 30)	Human (*n* = 16)	Serum and CCM of primary ESC isolated from endometriomas	Serum: ExoQuick Exosome Precipitation Solution kit (#EXOQ5A-1) CCM: Total Exosome Isolation Reagent (Thermo Fisher Scientific)	Partially	lncRNA aHIF	aHIF is elevated in serum EVs from patients with endometriosis compared to controls	Potential therapeutic target	aHIF is transferred from endometrioma stromal cells via EVs to stimulate angiogenesis	Endometriomas only. Methods suggest FBS in culture media is not EV depleted, introducing contaminating EVs
Qiu 2020 [35]	Human (*n* = 29 serum; *n* = 10 endometrial cells)	Human (*n* = 16 serum; *n* = 10 endometrial cells)	Serum, CCM of primary ESCs isolated from endometriomas	Serum: ExoQuick Exosome Precipitation Solution kit (#EXOQ5A-1; SBI). CCM: Total EVs isolation reagent kit (#44,578,259; Thermo Fisher Scientific)	Yes	lncRNA TC0101441	Potential diagnostic Serum TC0101441 increases with stage	Potential therapeutic target	EV TC0101441 may promote invasion and migration by regulating N-cadherin, snail, slug and TCF8/ZEB1	Endometriomas only
Shan 2021 [36]	Human (*n* = 5 for RNA seq, *n* = 80 for validation)	Human (*n* = 6 for RNA seq, *n* = 80 for validation)	Serum	ExoRNeasy Serum/Plasma Midi Kit (#76,214, Qiagen)	Yes	lncRNAs (seq) Ten lncRNAs validated with RT-qPCR	Potential diagnostic marker RP3-399L15.2 AUC 0.86, Sn 67%, Sp 98%. Using combination of RP3-399L15.2 and CH507-513H4.6 AUC 0.90, Sn 80%, Sp 85%.	Not discussed, but potential therapeutic targets	Not discussed	Endometriomas only
Sun 2018 [37]	Mouse (*n* = 10)	Mouse (*n* = 10)	ESC culture media	UC	Yes	Not investigated	Not discussed	Not discussed, potential therapeutic target	Endometriosis derived EVs induce M2 macrophage phenotype. In vivo, treatment with endometriosis EVs increased lesions.	EV cargo not investigated, no proposed mechanism of action
Sun 2019 [38]	Human (*n* = 22)	Human (*n* = 6)	CCM of primary ESCs isolated from endometrium of patients with and without endometriosis	UC	Yes	Not investigated	Not discussed	Not discussed, potential therapeutic target	ESC EVs from patients with endometriosis induce angiogenesis, neurite outgrowth and inhibit neuron apoptosis	In vitro only
Sun 2021 [39]	Human (*n* = 52)	Human (*n* = 21)	CCM of ESCs from ovarian endometrial cyst walls, and serum	ExoQuick EV precipitation solution kit (#EXOTC50A-1; SBI)	Yes	LGMNP1	Elevated serum EV LGMNP1 associated with infertility and recurrence. AUC for serum EV LGMNP1 to predict recurrence 0.869. Sn 93.3% and Sp 75.7%	Not discussed, potential therapeutic target	Endometriosis ESC EVs induce macrophage M2 phenotype via transfer of LGMNP1	Cyst wall derived ESCs only, may not be reproducible. Methods suggest FBS in culture media is not EV depleted, introducing contaminating EVs
Texido 2014 [40]	Human (*n* = 14)	Human (*n* = 13)	Fluid from simple cyst or endometrioma	ExoQuick Exosome Precipitation Solution (SBI)	Partially	Ectonucleotidases	Not discussed	Not discussed	Endometrioma EVs had increased ectonucleotidase activity compared to control	Cyst fluid only
Wu 2018 [41]	Human (*n* = 10)	None for EV experiments	CCM of primary ESCs from endometriomas	UC	Partially	miR-214	Not discussed, potential for diagnosis	Therapeutic potential of EV miR-214	MiR-214 enriched EVs down regulated CTGF and collagen (fibrosis) in a mouse model (*n* = 4)	Endometriomas only
Wu 2020 [42]	Human (*n* = 3 for seq, *n* = 7 for validation)	Human (*n* = 3 for seq, *n* = 7 for validation)	CCM of primary ESCs from paired endometriomas and endometrium, and endometrium from controls	ExoQuick-TC Exosome Isolation Kit (SBI)	Yes	lncRNA, miRNA and mRNAs (seq). Expression of regulatory networks LOC105376166/ miR-214-3p/ MIB2 and LOC105371414/ miR-423-5p/ADCY3 validated with RT-qPCR	Possible use of panel of EV derived RNA processing for diagnosis	Not discussed	Not discussed	Very large number of multiple comparisons with small subject numbers
Wu 2021 [43]	Human (*n* = 42)	Human (*n* = 24)	Serum	Beads/Precipitation	Partially	miRNAs (miRNA chip) miR miR-26b-5p, miR-215-5p and miR-6795-3p validated with RT-qPCR	miR-26b-5p and miR-215-5p were decreased, and miR-6795-3p was increased in endometriosis, and related to stage	Not discussed	MiRNAs identified may be involved in MAP and PI3K-AKT signalling.	No reporting on miRNA chip analysis methods
Wu 2021 [44]	Human (RNA seq *n* = 3, RT-qPCR *n* = 10)	Human (RNA seq *n* = 3, RT-qPCR *n* = 10)	CCM of primary ESCs from paired endometrioma and endometrium, and control endometrium	ExoQuick-TC Exosome Isolation Kit (SBI)	Yes	circRNAs, miRNAs, mRNAs (seq). circ_0026129, miR-15a-5p and ATP6V1A validated with RT-qPCR	Network analysis of ceRNAs lead to identification of three central components different between subjects and controls (circ_0026129, miR-15a-5p and ATP6V1A), possible diagnostic targets	Potential diagnostic targets	Increased miRNA-15a-5p known to be involved with angiogenesis. ATP6V1A likely involved with cell migration and growth.	Small numbers. Low statistical power with large number of comparisons and complex modelling calculations
Zhang 2020a [45]	Human (discovery cohort *n* = 5, validation *n* = 20)	Human (discovery cohort *n* = 5, validation *n* = 20)	Serum	UC	Yes	MiRNAs (microarray). RT-qPCR validation of miR-22-3p and miR-320a	miR-22-3p AUC was 0.855 (*p* < 0.01), miR-320a AUC was 0.827 (*p* < 0.01), combined AUC was 0.883 (*p* < 0.01). MiR-22-3p associated with high stage.	Not discussed	miR-22-3p and miR-320a increased in endometriosis, may be associated with MAP and TNF signalling.	Limited numbers
Zhang 2020b [46]	Human (*n* = 20)	Human (*n* = 20)	CCM of primary macrophages isolated from peritoneal fluid	UC	Yes	MiRNAs (microarray). RT-qPCR validated increased miR-22-3p in patient EVs	Not discussed	MiR-22-3p/SIRT1/NFkB pathway could be novel therapeutic target	Endometriosis macrophage derived EVs promote proliferation, migration and invasion of ESCs compared to macrophage EVs from benign controls via delivery of miR-22-3p. MiR-22-3p activates NF-kB via SIRT1.	Number of biological replicates for cell culture assays unclear. Methods suggest FBS is not EV depleted, introducing contaminating EVs
Zhang 2021 [47]	Human (serum *n* = 20)	Human (serum *n* = 20)	CCM of primary ESCs derived from endometriomas and control endometrium, and serum	ExoQuick-TC Exosome Isolation Kit (SBI)	Partially	miR-214-3p	MiR-214-3p decreased in serum of endometriosis patients (*p* < 0.05).	EV miR-214-3p treatment reduced fibrosis in mice.	Downregulation of miR-214-3p increases fibrosis in vitro and in vivo via CCN2.	N for primary cultures unclear. Endometriomas only. Authors report serum miR-214-3p is from EVs however their methods indicate it is total plasma RNA, not EV specific.
Zhou 2020 [20]	Human (*n* = 3)	Human (*n* = 3)	CCM of primary ESCs from endometrium of women with endometriomas and infertility, and fertile controls	ExoQuick-TC Exosome Isolation Kit (SBI)	Yes	Small RNAs (seq)	Potential diagnostic	Not discussed	Target gene prediction identified *HOXA10* and *LIF* as possible targets Enriched pathways included MAPK and Wnt signalling	Low numbers, no validation of RNA seq via RT-qPCR

*AUC* Area under the curve, *CCM* Conditioned culture media, *ceRNA* Competing endogenous RNA, *DIE* Deep infiltrating endometriosis, *ESCs* Endometrial stromal cells, *EV* Extracellular vesicle, *FBS* Fetal bovine serum, *lncRNA* Long non-coding RNA, *miRNA* Micro-RNA, *mRNA* Messenger RNA, *MSCs* Mesenchymal stem cells, *RT-qPCR* Quantitative reverse transcription polymerase chain reaction, *SBI* System Biosciences, *SEC* Size exclusion chromatography, *seq* Sequencing, *Sn* Sensitivity, *Sp* Specificity, *UC* Ultracentrifugation

^a^EV characterisation requirements as outlined in the minimal information for studies of extracellular vesicles (MISEV2018) guidelines

## Results

### Search results

**Fig. 1:**
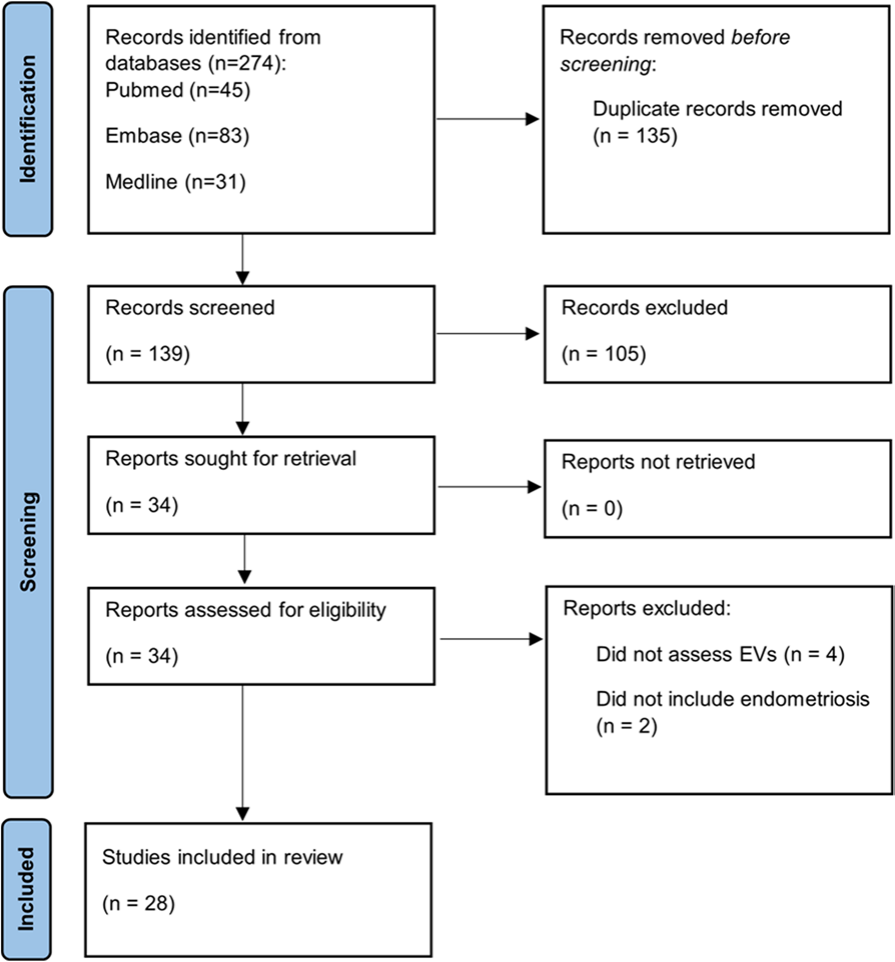
Identification of studies via databases and register

Twenty eight studies were included in the review. The initial search identified 274 articles of which 139 were unique. When applying inclusion criteria, 105 of these studies were excluded. A further six studies were excluded as they either did not specifically state that EVs were assessed, or did not specifically state that subjects had endometriosis. The flow of study identification is shown in Fig. 1.

### Terminology

For the purposes of this review we use the term “endometrium” to imply eutopic endometrium; “endometriosis lesions” to describe any lesions outside the endometrium (commonly described as “ectopic endometrium” in the cited studies) and “endometrioma” to describe any endometriotic cysts on the ovary.

Much of the current EV literature uses inconsistent nomenclature, naming a subtype of EVs such as exosomes without demonstrating the biogenic origins of the EVs in the fractions isolated. Thus, this review will use the term ‘EV’ throughout.

### Source of extracellular vesicles

Endometriosis related EVs were isolated from peritoneal fluid [18, 23, 27, 28, 30, 33, 46], endometrium [18–20, 25, 28, 29, 34, 35, 37, 38, 41, 42, 44, 47], endometriosis lesions [18, 19, 26, 28], endometriomas [25, 30, 34, 35, 39–42, 44, 47], cervicovaginal swabs (in a single monkey) [32], plasma [18, 31] and serum [18, 26, 28, 31, 34–36, 39, 43, 45, 47]. (Plasma is recommended by the ISEV over serum, due to the release of a significant amount of additional EVs from platelets during the clot formation when preparing serum [48]). Additionally, one study reported on EVs from umbilical cord derived stem cells, which were used in vitro as a potential therapeutic agent [24]. EVs were isolated from the conditioned media of primary ex vivo cultures in sixteen studies [19, 20, 24, 25, 28–30, 34, 35, 37, 38, 41, 42, 44, 46, 47].

### Isolation and identification of extracellular vesicles

Isolation techniques varied between studies. Ultracentrifugation (UC) [19, 23, 25–27, 29, 30, 32, 37, 38, 41, 45, 46] and commercial kits [18, 20, 24, 28, 34–36, 39, 40, 42–44, 47] were the most commonly used methods (each used in thirteen studies) while two used size exclusion chromatography (SEC) [28, 33] and one was not reported [31]. Fifteen of twenty-eight studies (53%) fully characterised EVs according to the MISEV2018 guidlines, with the majority utilising western blot, electron microscopy and nanoparticle tracking analysis. Ten studies (36%) completed partial characterisation (did not fulfill all guidelines) and three (11%) did not report any EV characterisation.

### Cargo and function of extracellular vesicles

**Fig. 2:**
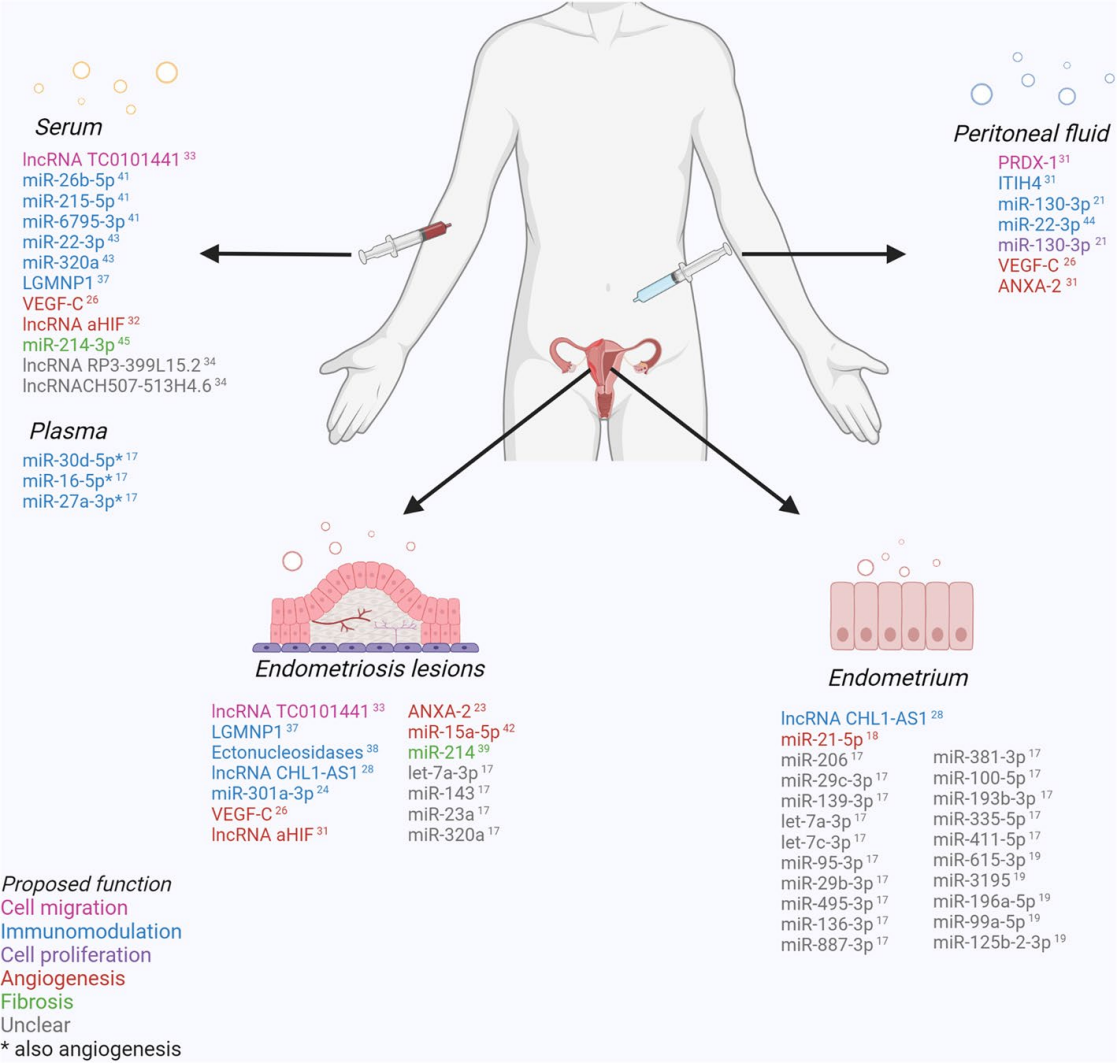
Extracellular vesicle cargo identified in subjects with endometriosis from different sites, with proposed function

The cargo of EVs were studied for their potential diagnostic utility, therapeutic utility or as a key element of the pathogenesis for endometriosis. Cargo studied include miRNAs involved with immunomodulation and cell proliferation [18, 20, 23, 43, 45, 46], angiogenesis [18, 19, 44] and fibrosis [41, 47]; proteins involved with angiogenesis [24, 28] and cell proliferation [25, 33]; lncRNAs involved with immunomodulation [30, 36], cell proliferation [36], cell migration [30, 35, 36] and angiogenesis [34]; and other targets including pseudogene LGMNP1 [39] and ectonucleotidases [40]. The specific cargo and their site of origin are listed in Fig. 2.

### Pathophysiology

Proposed roles of EV cargo in the pathogenesis of endometriosis are outlined in Fig. 2. Biopsies from the endometrium showed differences in EV cargo between people with and without endometriosis in five studies [18–20, 30, 42]. These included cargo involved in immunomodulation [30] and angiogenesis [19], and multiple miRNAs with as yet unclear function [18, 20]. Network analysis showed different expressions of miRNA, lncRNAs and mRNAs between groups [42].

EVs isolated from peritoneal fluid also showed differences between people with and without endometriosis in three studies [23, 33, 46]. Specifically, cargo were identified that play a role in cell migration [33], immunomodulation [23, 33, 46], cell proliferation [23] and angiogenesis [23, 33].

EVs isolated from endometriotic lesion biopsies contained cargo involved with cell migration [35], immunomodulation [26, 30, 39, 40] and angiogenesis [25, 28, 34, 44]. Several miRNAs with unclear function were also found to be present in levels significantly different from endometrial and peritoneal fluid samples in controls [18].

EVs isolated from plasma or serum showed differences between people with and without endometriosis in eight studies [18, 28, 34–36, 43, 45, 47]. Specifically, cargo were identified that play a role in cell migration [35], immunomodulation [18, 39, 43, 45], angiogenesis [18, 28, 34], fibrosis [47] and several lncRNAs with unclear function [36].

In addition, one study showed that EVs derived from mesenchymal stem cells may enhance migration of endometrial cells and therefore play a part in endometriosis [24]. Finally, one study showed differences in EVs derived from microbes in peritoneal fluid between women with endometriosis and controls, suggesting a role for microbiome in endometriosis aetiology [27]. The microbiome has been previously postulated to have an important role of the pathophysiology of endometriosis [21, 49]; EVs may provide a powerful tool to explore this further.

### Diagnostic markers

Three studies reported on sensitivity and specificity of specific EV cargo as biomarkers [28, 36, 45]. One study of 48 patients and 21 controls reported serum EV derived VEGF-C as a biomarker with sensitivity 81.3% and specificity 71.4% for endometriosis [28]. Another study of 85 patients and 85 controls reported serum EV derived lncRNA RP3-399L15.2 as a biomarker with sensitivity 67% and specificity 98%; or using a combination of lncRNAs RP3-399L15.2 and CH507-513H4.6 a sensitivity of 80% and specificity of 85% [36]. A third study of 20 subjects and 20 controls reported a combination of serum EV derived miRNAs 320a and 22-3p as having sensitivity of 80% and specificity of 80% [45].

Additionally, a study of 52 patients and 21 controls reported serum derived pseudogene LGMNP1 having sensitivity 93% and specificity 76% in predicting recurrence within the four year study period, with a significant difference between subjects with primary and recurrent disease [39].

### Extracellular vesicle derived therapeutic targets

Two studies utilised EVs derived from peritoneal macrophages as a potential therapeutic agent; demonstrating an ability to inhibit angiogenesis, migration and invasion of endometriosis in mouse models of endometriosis [29, 30]. One study utilised EV derived miR-214 to downregulate fibrosis in a mouse model of endometriosis [41]. Another study showed that EV derived miR-301a-3p was overexpressed in endometriosis lesions compared with serum from healthy controls; and that downregulation of this miRNA in a mouse model influenced macrophage activity [26].

### Limitations

Specific limitations for each study are listed in Table 1.

Out of the twenty-six studies using patient tissue, thirteen reported endometriosis stage using the revised American Society of Reproductive Medicine (rASRM) classification system [18, 20, 23, 27, 28, 33, 36, 38, 41–45], four used the American Fertility Society (AFS) classification system [31, 34, 35, 39], and the remaining nine did not report the disease severity in the patient population [19, 24–26, 29, 30, 40, 46, 47]. Nine studies only included participants with endometriomas [20, 25, 34–36, 41, 42, 44, 47].

Other common limitations included small sample sizes, use of fetal bovine serum (FBS) for culture media supplementation without prior EV depletion [34, 39, 46] and unclear number of participants or samples used for assays.

## Discussion

Whilst in its infancy, the use of EVs as diagnostic biomarkers or therapeutic targets in endometriosis has significant potential to improve the outcomes of people affected by this disease; additionally, several insights into the pathogenesis of the disease have been obtained. The recent excitement around EVs in cancer research has resulted in the development of strict reporting guidelines for researchers, with the next iteration of the MISEV guidelines to be released this year. As such the groundwork for investigating EVs in endometriosis is well established.

Using a systematic search protocol, we have identified and reviewed 28 studies that investigate EV cargo in endometriosis. Studies varied in their patient population, type of endometriosis, type of sample and EV isolation which created bias and difficulty in comparing studies; however, several insights into pathogenesis have been gained; diagnostic utility has been demonstrated; and therapeutic targets have been identified.

### Limitations

There are multiple limitations in the included studies, which we broadly divide into clinical population limitations and laboratory processing limitations.

The clinical populations included in these studies were heterogeneous; with few studies reporting subject age and symptoms, and 65% reporting laparoscopic diagnosis and disease severity using accepted scoring systems. The limitation of current endometriosis scoring systems, especially with regards to clinical application, is well established; however in a research setting accepted scoring systems give some insight into the pathological extent of disease [50]. Nine studies only included subjects with endometriomas, and it is unclear at present whether EVs in this setting would differ from other manifestations of endometriosis.

With regards to laboratory processing bias, adherence to established MISEV standards was inconsistently achieved, limiting the reproducibility and reliability of many of the included studies. Complete EV characterisation is highly recommended to provide evidence of EV involvement when proposing a role in pathogenesis or EV cargo as biomarkers. The challenge of EV research is the heterogeneity of EV isolation methodologies creates difficulty in making meaningful comparisons across studies. Isolation methodology impacts on the composition of the EV fraction, with different size and density EVs and co-isolated contaminants favoured [51]. The lack of overlapping targets identified in any of the studies reviewed is likely a reflection of the diverse combinations of EV source, isolation techniques and methodologies used. Study reproducibility and the ability to validate study results is hindered by the absence of standardised sample processing and EV isolation methods.

The majority (57%) of the listed studies isolated EVs from ex vivo cell culture of primary stromal, mesenchymal or endometriosis samples. Cell culture purity or validation of cell type in these studies were not performed. The digestion of tissue and 2D culturing of primary cells in a variety of media (EV depleted or not) has a significant impact on the EV and EV cargo that is released [52]. Therefore, it is difficult to compare EV isolation from primary cell culture to those found directly in patient samples.

### Diagnostic potential

There is a critical need for non-invasive diagnostic biomarkers in endometriosis. The lack of specific symptoms, limited understanding of the pathology of the disease and delay from symptom onset to diagnosis via invasive laparoscopy creates a serious clinical challenge in treating endometriosis. As cell signalling mediators which carry tissue/disease specific cargo, EVs hold the potential to be exploited as diagnostic biomarkers. EVs in peritoneal fluid and serum probably represent a combination of both diagnostic and prognostic categories; however, sampling peritoneal fluid is not particularly useful as a diagnostic tool as laparoscopy or other invasive procedures are required. Identifying the EV cargo derived from a combination of endometrium and endometriosis lesions in serum likely holds the greatest pragmatic potential as a non-invasive diagnostic biomarker and may provide insight into both “potential for disease” and “progression of disease”.

**Fig. 3:**
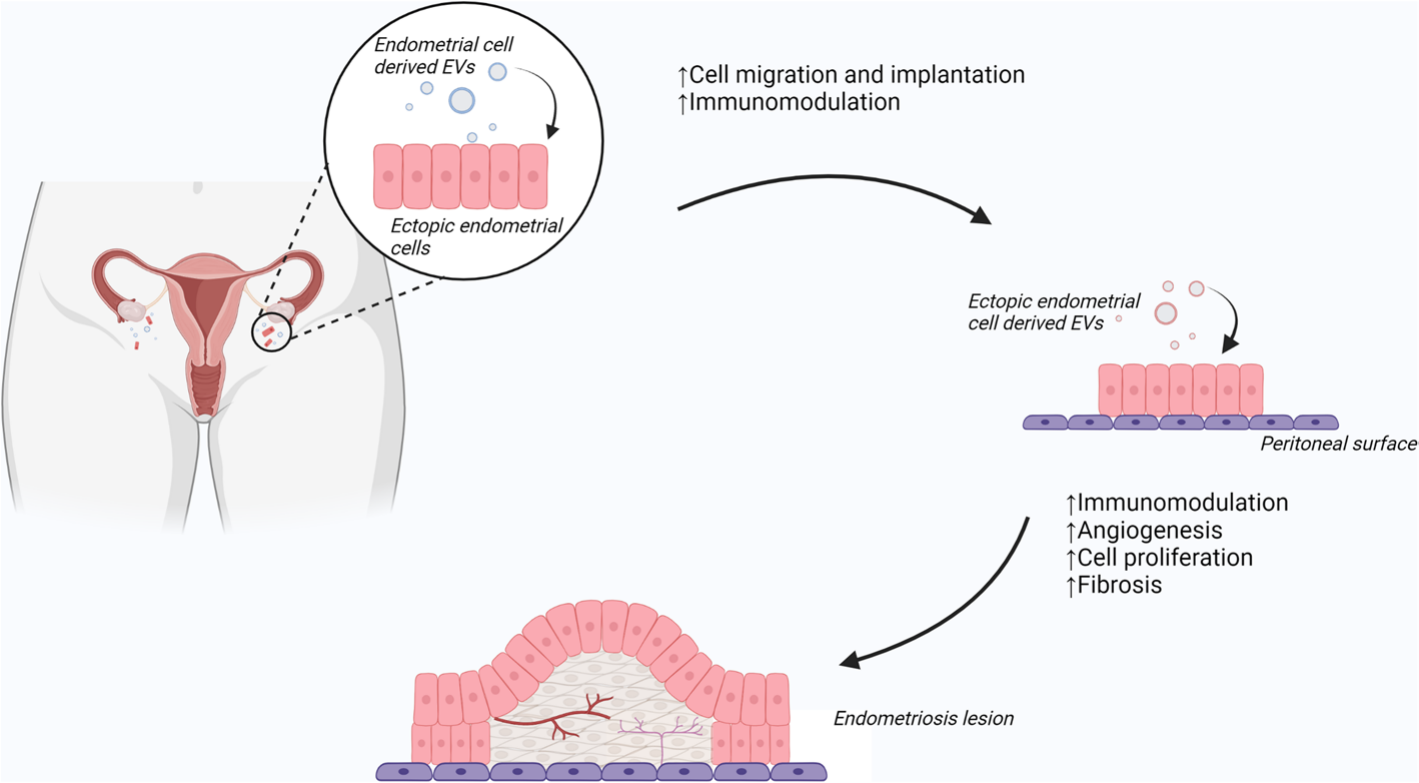
Proposed role of extracellular vesicles (EVs) in the development of endometriosis lesions. EVs derived from the endometrium act on endometrial cells shed during retrograde menstruation, allowing migration, implantation and immunomodulation. EVs from newly implanted ectopic endometrial cells enhance pathogenesis with ongoing immunomodulation and cell proliferation, as well as angiogenesis and fibrosis, leading to the formation of endometriosis lesions

Four studies demonstrated that in samples taken from the endometrium, differences could be identified between people with and without endometriosis [18–20, 30]. As shown in Fig. 3, these differences may imply a susceptibility to developing endometriosis as molecules within these EVs appear to facilitate the development of endometriosis lesions by influencing cell migration and proliferation and immunomodulation. These EVs may contribute to a phenotype susceptible to developing endometriosis, and are unlikely to be affected by surgical resection of disease, however this is speculative and yet to be investigated.

In contrast to this, EVs obtained from the peritoneal fluid and serum are likely to contain EVs derived from the endometrium in addition to EVs derived directly from endometriosis lesions, as well as numerous other sources. The diagnostic potential is therefore increased; however, reliable identification of the relevant EVs poses a more pronounced challenge. It could be assumed that analysis of EVs in these samples, in contrast to endometrial samples, would be markedly different after complete surgical excision of endometriosis; and therefore may bear promise as markers of current disease state. This could be exploited to detect recurrence, as was demonstrated in one small study [39].

Only 8 of the 28 studies systematically reviewed identified or discussed potential diagnostic markers, and only 4 of these measured AUC and/or sensitivity and specificity. These were serum RP3-399L15.2 [36] (Sn 67% and Sp 98%), serum VEGF [28] (Sn 81% Sp 71%), serum miR-22-3p and miR-320a [45] (AUC 0.883) and LGMNP1 [39] (as a marker for recurrence Sn 93% Sp 75%). Whilst promising, these studies have limited sample sizes (ranging from *n* = 20 to *n* = 86) and therefore further investigation and validation is required before clinical utility can be addressed.

Validation of these candidate diagnostic markers using clinically relevant means of isolation and quantification are also required. miR-22-3p and miR-320a were measured as biomarkers in EVs isolated using UC, while SEC was utilised when investigating EV-VEGF. While UC is the still the most widely used EV isolation methodology [53] and SEC generally yields higher purity EV fractions (depending on biofluid) [54], both have limited clinically utility due to specialist equipment requirements, typically time-consuming protocols and lack of scalability. Commercial kits tend to have short, straightforward protocols that are appealing for clinical use, however are plagued with high levels of non-EV contamination [55]. Therefore, the ideal means of EV isolation for clinical use may not yet have been developed; candidate biomarkers identified thus far may require future validation with a clinically implementable test.

### Therapeutic potential

Many of the studies in this review used ex vivo cell culture techniques to investigate EV function in mediating macrophage polarisation, angiogenesis or proliferation. Therefore, the EV cargo identified in these studies were deemed potential therapeutic targets as they had some impact on endometriosis growth and inflammation.

Four studies using ex vivo cell culture techniques and mouse models were able to demonstrate disruption to the pathogenesis of endometriosis by targeting EVs therapeutically [26, 29, 30, 41]. As discussed above, the effect of ex vivo cell culture on EVs limits the reliability of such research; nonetheless there is promise and a myriad of potential targets for ongoing research to develop therapeutic agents. Significant advances need to be made before use in the clinical setting becomes conceivable.

If there is indeed a specific EV phenotype that facilitates development of endometriosis, it is plausible that therapeutic agents aimed at cargo of these specific EVs may be able to prevent disease or recurrence; however, the utility in treating already established lesions may be limited.

### Recommendations for future research

As the diagnostic and therapeutic potential for endometriosis from EV research increases; we expect to see a larger focus on clinical aspects in future studies. Endometriosis is a vastly heterogeneous disease, with clinical symptoms ranging from asymptomatic through to severely debilitating; and surgical disease ranging from minimal to major pathological anatomical changes, with limited correlation between the two [56]. Detailed clinical data on study participants, their symptomatology and surgical data would add further understanding into how EVs differ across this incongruous disease spectrum.

While several studies have shown promising diagnostic potential for the use of EVs as a non-invasive diagnostic biomarker; validation of these studies in large populations is warranted; additionally, more detailed comparison between subjects with differing clinical and surgical severity would add further insight into the clinical utility of such tests. Future studies into diagnostic biomarkers should focus on using clinically accessible EV isolation methods, to maintain the relevance and translatability of the study.

The effect of surgical excision on endometriosis associated EVs is yet to be established. As outlined above, we hypothesise that some endometrium derived EVs may predispose to endometriosis, and that these would not be affected by surgical intervention; whilst EVs derived from endometriosis lesions would diminish significantly after surgery and therefore potentially act as a marker of recurrence. Only one study looked at recurrence [39] and would support this hypothesis, however, studies to specifically address this issue would provide further insight.

As shown in Fig. 2 above, there was very little crossover between identified EV targets across the 28 studies included in this review; and currently there are hundreds if not thousands of specific identified EV cargo from multiple bodily fluids that play a role in endometriosis. With such a wide number of comparisons across studies and relatively small participant numbers, it is likely that some false positives exist. There may be merit in future studies further validating currently identified targets rather than searching for new targets, such that meta-analyses may be performed in the future to bolster subject numbers. Identifying similar targets across multiple different bodily fluids may also provide further insight into their pathological relevance and clinical utility.

Finally, ongoing research into potential therapeutic EV targets is currently limited to animal models; however, as identification of relevant EV cargo and their pathological roles continues, further research into the disruption of endometriosis pathogenesis by targeting these EVs may provide the pathway for non-surgical non-hormonal treatment options.

## Conclusion

Endometriosis is a chronic, inflammatory gynaecological disease that can have severe impacts on quality of life, placing burden on patients and the healthcare system. Due to the heterogeneous nature of endometriosis, diagnosis and treatment remain a significant clinical challenge. EVs are a relatively new field of biomedical research and have potential to be used as diagnostic tools in a range of diseases. EVs contribute to cell migration, implantation and immunomodulation in endometriosis; this can likely be exploited for diagnostic and prognostic information and may provide new therapeutic targets. We encourage further EV research in endometriosis to include rigorous reporting of both the patient population and technical methodology, with the goal of achieving clinical utility for diagnosis, prognosis and eventually treatment.

## Supplementary Information

**Additional file 1.**

## Data Availability

Not applicable.

## Abbreviations

AFS: American Fertility Society
AUC: Area under the curve
EV: Extracellular vesicle
FBS: Fetal bovine serum
ISEV: International Society of Extracellular Vesicles
lncRNA: Long non-coding RNA
miRNA: Micro RNA
MISEV: Minimal information for studies of extracellular vesicles
mRNA: Messenger RNA
rASRM: Revised American Society of Reproductive Medicine
SEC: Size exclusion chromatography
UC: Ultracentrifugation
