# Supplementary material for: A promising future for endometriosis diagnosis and therapy: extracellular vesicles - a systematic review
Source: Reprod Biol Endocrinol. 2022 Dec 21;20:174. doi: 10.1186/s12958-022-01040-y (PMC9768904; doi:10.1186/s12958-022-01040-y)
Supplement: Supplementary file 1 — Additional file 1. [file 12958_2022_1040_MOESM1_ESM.docx]

**Appendices**

Methods

Search was performed on 24/3/22 as per the following protocol.

**Pubmed (45 results)**

("extracellular vesicles"[MeSH Terms] OR extracellular vesicle[Text Word] OR "exosomes"[MeSH Terms] OR exosome[Text Word]) AND ("Endometriosis"[Mesh] OR endometriosis[Text Word])

**Embase (83 results)**

Endometriosis/

Extracellular vesicles.mp. or exosome/

1 and 2

**Medline (31 results)**

Endometriosis/

Extracellular Vesicles/

Exosomes/

2 or 3

1 and 4

**Web of Science (65 results)**

TS=(endometriosis) AND ((TS=(extracellular vesicles)) OR TS=(exosomes))

**Google Scholar (2980 results - limit to 50 by relevance)**

"endometriosis" AND ("extracellular vesicles" OR "exosomes")
